# Supplementary material for: Comparative analysis of experimental and numerical investigation on multiple projectile impact of AA5083 friction stir welded targets
Source: PLoS One. 2023 Jul 27;18(7):e0285254. doi: 10.1371/journal.pone.0285254 (PMC10374147; doi:10.1371/journal.pone.0285254)
Supplement: Attachment — Submitted filename: comments (5).docx [file pone.0285254.s001.docx]

07.02.2023

Dear Prof. (Dr.) Editor-in-chief,

Thank you for considering the manuscript entitled, **“Experimental and numerical investigation of an AA5083 targets against multiple projectile impact: A comparative analysis”,** for the publication in **PLOS ONE**. I am grateful to you and the reviewers for the valuable suggestions provided. I like to resubmit our revised version of the manuscript by adding response to all your comments. Below please find the answers and actions taken to address these comments. All the suggestions are incorporated and highlighted with the **BLUE COLOR** in manuscript.

**NOTE:** All the necessary changes/added sentence has been shown in the **BLUE COLOR.**

The locations of these changes have been mentioned, where possible, in the action points that respond to each reviewers’ comments. Here are the responses to the reviewer comments:

**AUTHOR RESPONSE TO REVIEWER AND EDITOR COMMENTS**

**Manuscript Number:** PONE-D-22-32018

**Article title:** Experimental and numerical investigation of an AA5083 targets against multiple projectile impact: A comparative analysis

**Journal:** PLOS ONE

The manuscript has been thoroughly modified and improved the quality of the content to meet the standards of the Journal. All the suggestions made by the learned referees are included in the revised manuscript. We are extremely thankful to the referees & editor(s) for their constructive comments and appreciation.

**Response to Reviewer’s Comments**

*The authors are grateful to the reviewers for their suggestions that have all contributed to improving the manuscript. Once again, the authors are extremely thankful for the observations and the comments of the reviewers. All the comments are appropriately addressed and now the quality of the article has been appreciably enhanced before the consideration for publications. The rebuttal file is enclosed indicating the revisions incorporated in the article as suggested. The revisions are carried out in* **BLUE COLOR** *in the text of the manuscript for better visibility to the reviewers and as well as to the editor.* *We have made the modifications as per their suggestions in the revised manuscript and changes are also marked up using the* “**BLUE COLOR**” *function.*

*All in all, the authors should thank the reviewers for their meticulous observations in reviewing the article. All the issues raised by the authors are appropriately addressed as stated in the following table,*

**Author Response to Reviewer’s Queries**

We express our sincere thanks to the reviewers and editor for their valuable suggestions to improve our manuscript. The following are the detailed author’s response (AR) for reviewer’s queries (RQ).

**Reviewer #1: Queries and authors response**

The authors perform experimental and numerical work on AA5083 against ballistic impact. The work should be improved for publication.

| RQ:1 | The description of the behavior of AA5083 is quite imprecise. The JC Model is a thermoviscoplastic model to model the behavior of the material over a wide range of temperatures and strain rates. It should be rewritten. The use of "" is not understood in very clear terms. |
| --- | --- |
| AR:1 | **As per reviewer comment, the above statement has been rewritten.** |
| RQ:2 | Figure 2 is dispensable. In addition, the following sentence is included in the parenthesis: "The heat input in the welded region was 322W [27] and it was given to all over the weld region". This has been modeled? |
| AR:2 | **As per reviewer comment, Figure 2 has been removed and heat input was taken from the reference [27]. During simulation heat input value is assigned in stir zone region.** |
| RQ:3 | Have they used a damage initiation and evolution model in ABAQUS or was it a Shear Failure? |
| AR:3 | **We have used Johnson-Cook material and damage model in ABAQUS.** |
| RQ:4 | The mesh in Figure 6 looks overly extorted. |
| AR:4 | **Based on mesh convergence study, we have arrived the mesh size. Hence it was extorted.** |
| RQ:5 | The concepts of damage mechanisms are wrong. |
| AR:5 | **The concepts of damage mechanisms have thoroughly been explained based on common type of ballistic failure mechanisms.** |
| RQ:6 | The paper is quite confusing, it should start with the experimental part |
| AR:6 | **As per reviewer comment, the order has been changed. The authors have tried to refine the overall content, structure, formatting, and relevancy of the revised article, specifically in the Introductions section as per the learnt recommendations. The authors have now cited a number of relevant recent articles on related works, thereby enhancing the content's appeal and retainability among readers of scientific journals. In the revised article, the authors have attempted to refine the overall content, structure, scientific contributions, novelty, and relevance as per the learned recommendations.**  **Additionally, the authors have removed any unnecessary or undesirable information in the article and polished the manuscript in a more accurate and precise way, removing any junk content, and organizing it in an orderly and systematic format related to the literature studies, research gaps, problem formulation, objectives, and research methodology. By systematically supporting the present results with previous literature reviews, the outlines of the discussions have been extensively expanded and furthermore enumerated in a more comprehensive and extensive way.**  **All in all, the authors have excavated or removed the unnecessary matter or data from the article and thus, the manuscript has been refined, prepare and contemplate it in more precise or accurate, and thus, organized in the systematic orderly manner.** |

**Reviewer #2: Queries and authors response**

| RQ:1 | In my opinion, no new finding can be found in the present study. Therefore, It should be rejected. The other reason is that the content of to present study is not well fitted with the aim and scope of the Journal. |
| --- | --- |
| AR:1 | **The authors are enormously thankful to the learnt referee for their knowledgeable insights on the same. The novelty statement and scientific contributions have extensively been enumerated in revised manuscript as exhibited in the blue colour.**  **The authors have tried to refine the overall content, structure, formatting, and relevancy of the revised article, specifically in the Introductions section as per the learnt recommendations. The authors have now cited a number of relevant recent articles on related works, thereby enhancing the content's appeal and retainability among readers of scientific journals. In the revised article, the authors have attempted to refine the overall content, structure, scientific contributions, novelty, and relevance as per the learned recommendations.**  **Additionally, the authors have removed any unnecessary or undesirable information in the article and polished the manuscript in a more accurate and precise way, removing any junk content, and organizing it in an orderly and systematic format related to the literature studies, research gaps, problem formulation, objectives, and research methodology. By systematically supporting the present results with previous literature reviews, the outlines of the discussions have been extensively expanded and furthermore enumerated in a more comprehensive and extensive way.** |

**Reviewer #3: Queries and authors response**

| RQ:1 | In the Title FSW title should be included |
| --- | --- |
| AR:1 | **As per reviewer comment, the friction stir welding has been added in the title of the paper.** |
| RQ:2 | Replace the term Base metal with Base material |
| AR:2 | **As per reviewer comment, the base metal has been replaced with base material.** |
| RQ:3 | “It was determined that weldments perform less well (30%) as compared to BM targets” reason can be included in the abstract |
| AR:3 | **As per reviewer comment, the reason for less ballistic performance of weldments has been included in the abstract** |
| RQ:4 | Scale bar missing in the Fig.3 |
| AR:4 | **As per reviewer comment, the scale bar has been added in the Figure 3** |
| RQ:5 | What are the process parameters considered for the current study? |
| AR:5 | **To obtain defect-free weld connections, a tool rotation speed of 680 rpm and a welding speed of 146 mm/min were used [27].**  **This are the process parameter used for preparation of weldments** |
| RQ:6 | Many typo errors are there in manuscript please check. |
| AR:6 | **As per reviewer comment, in the revised manuscript all typo errors has been corrected. As per suggestions, all the mistakes in accordance with the writing skills and usage of English language have now been polished up to a fervent extent with the assistance of the native English contributor/co-author of this article.**  **As per valuable suggestions received, the grammatical errors have now been removed and many sentences have been reframed so as to deliver clear interpretation in the revised manuscript.** |
| RQ:7 | For armoured vehicles require thicker section base material. But current study was lesser thickness |
| AR:7 | **Less thickness plates are used for fabrication of trailer beds in the defence tankers. Hence, our Initially study is focused on the lesser thickness plates.** |
| RQ:8 | Reframe article to publication format it looks like a report |
| AR:8 | **As per reviewer comment, the complete articles has been reframed. Now, the manuscript has now been restructured, refined, formatted, and extensively modified or revised as per the standard template of the Journal (PLOS ONE).**  **The authors have tried to refine the overall content, structure, formatting, and relevancy of the revised article, specifically in the Introductions section as per the learnt recommendations. The authors have now cited a number of relevant recent articles on related works, thereby enhancing the content's appeal and retainability among readers of scientific journals. In the revised article, the authors have attempted to refine the overall content, structure, scientific contributions, novelty, and relevance as per the learned recommendations.**  **Additionally, the authors have removed any unnecessary or undesirable information in the article and polished the manuscript in a more accurate and precise way, removing any junk content, and organizing it in an orderly and systematic format related to the literature studies, research gaps, problem formulation, objectives, and research methodology. By systematically supporting the present results with previous literature reviews, the outlines of the discussions have been extensively expanded and furthermore enumerated in a more comprehensive and extensive way.**  **All in all, the authors have excavated or removed the unnecessary matter or data from the article and thus, the manuscript has been refined, prepare and contemplate it in more precise or accurate, and thus, organized in the systematic orderly manner.** |

Hence, a scientific explanation of the obtained results has been refined and ameliorated up to a fervent extent. Results are enumerated, test methods are utterly described, interpretation have been corelated with results and previous literature findings. The overall summary should indicate the progress of the research and the limitations.

**Note: All the necessary changes/added sentence has been shown in the BLUE COLOR.**

Thank you very much in advance for taking your time in reviewing this manuscript.

Sincerely, we hope you will find our revision satisfactory.

Thanks, in anticipation.

Regards,

Dr. Shubham Sharma

(Corresponding author)
