# Supplementary material for: Comparative analysis of experimental and numerical investigation on multiple projectile impact of AA5083 friction stir welded targets
Source: PLoS One. 2023 Jul 27;18(7):e0285254. doi: 10.1371/journal.pone.0285254 (PMC10374147; doi:10.1371/journal.pone.0285254)
Supplement: Attachment — Submitted filename: comments (6).docx [file pone.0285254.s002.docx]

02.04.2023

Dear Prof. (Dr.) Editor-in-chief,

Thank you for considering the manuscript entitled, “**Experimental and numerical investigation of an AA5083 friction stir welded targets against multiple projectile impact: A comparative analysis**”, for the publication in **PLOS ONE**. I am grateful to you and the reviewers for the valuable suggestions provided. I like to resubmit our revised version of the manuscript by adding the response to all your comments. Below please find the answers and actions taken to address these comments. All the suggestions are incorporated and highlighted in **BLUE COLOUR** in the manuscript.

NOTE: All the necessary changes/added sentence has been shown in the **BLUE COLOUR**.

The locations of these changes have been mentioned, where possible, in the action points that respond to each reviewers’ comments. Here are the responses to the reviewer comments:

**AUTHOR RESPONSE TO REVIEWER AND EDITOR COMMENTS**

Manuscript ID: PONE-D-22-32018R1

Paper title: Experimental and numerical investigation of an AA5083 friction stir welded targets against multiple projectile impact: A comparative analysis

The manuscript has been thoroughly modified and improved the quality of the content to meet the standards of the Journal. All the suggestions made by the learned referees are included in the revised manuscript. We are extremely thankful to the referees & editor(s) for their constructive comments and appreciation.

**Response to Reviewer’s Comments**

The authors are grateful to the reviewers for their suggestions which have all contributed to improving the manuscript. Once again, the authors are extremely thankful for the observations and the comments of the reviewers. All the comments are appropriately addressed and now the quality of the article has been appreciably enhanced before the consideration for publication. The rebuttal file is enclosed indicating the revisions incorporated in the article as suggested. The revisions are carried out in **BLUE COLOUR** in the text of the manuscript for better visibility to the reviewers as well as to the editor. We have made the modifications as per their suggestions in the revised manuscript and changes are also marked up using the “**BLUE COLOUR**” function.

All in all, the authors should thank the reviewers for their meticulous observations in reviewing the article. All the issues raised by the authors are appropriately addressed as stated in the following table,

**Author Response to Reviewer’s Queries**

We express our sincere thanks to the reviewers and editor for their valuable suggestions to improve our manuscript. The following are the detailed author’s response (AR) for reviewer’s queries (RQ).

**Reviewer #1: Queries and authors response**

| RQ:1 | I am still unclear about the damage model they have used. If you refer to the ABAQUS documentation or any article (<https://abaqus-docs.mit.edu/2017/English/SIMACAEMATRefMap/simamat-c> johnsoncook.htm), you can see if they have used a damage initiation and damage evolution model. The damage evolution depends on a displacement/length parameter. |
| --- | --- |
| AR:1 | **The Johnson cook damage initiation and damage evolution model depends on following parameters [23-25]**   1. **Equivalent plastic strain, 2. D1 – D5 are material parameters 3. Strain-hardening constant 4. Thermal-softening constant 5. stress triaxiality ratio.**   **In addition, the study utilized the ABAQUS finite element code to conduct numerical simulations on a 3D structural FE model of AA5083 target's behavior upon projectile impact. The projectile was modeled as a rigid object, while the target was modeled as a deformable object. The study utilized the Johnson-Cook model to characterize the material behavior of the AA5083 target under the impact loading, and the damage model was used to determine the failure of the target when the damage parameter, D, reached unity. The study used mesh convergence studies to establish the best mesh size for the current situation. The results of the study showed that the target's damage parameters increased with an increase in the projectile's velocity, which ultimately led to the target's failure.**  **Furthermore, the numerical analysis of the impact of a projectile on a target was conducted using ABAQUS software. The target was modeled as a deformable object, and the projectile was modeled as a rigid object. The Johnson-Cook Model was used to model the material behavior of the target, considering factors such as strain hardening, plastic flow, thermal softening, and fracture. The failure of the target occurs when the damage parameter, D, reaches unity. Mesh convergence research was conducted to establish the best mesh size for the analysis. The model for the aluminum plate was built using around 9345 C3D8R hexahedral elements, and the model for the bullet was built with 2207 tetrahedral elements.** |
| RQ:2 | In Figure 8b and 8c, there is an element distortion problem. The element may have been deformed by 300% of its original size, and it should have been removed much earlier. |
| AR:2 | **As a result of this finding, there was clear deformation of the plate against the projectile penetration. This was the reason why we took the deformation image for so long, since it had been a while.** |
| RQ:3 | Spalling usually occurs in very high-strength steels with a large thickness. When spalling occurs, fragments are produced on the backside of the plate. I do not see such fragments in Figure 8. It appears that the projectile may have completely perforated the plate. |
| AR:3 | **We sincerely thank the reviewer for identifying the error that was made by us during the identification of ballistic failure mechanism. The type failure that occurred in the Figure 8 was ductile hole failure. The above correction has been updated in the revised manuscript.** |

**Reviewer #2: Queries and authors response**

| RQ:1 | Authors can Include latest journals related ballistic behavior of friction stir welded aluminum joints in the introduction. |
| --- | --- |
| AR:1 | **As per reviewer comment, few of the latest journals related to ballistic behavior of friction stir welded aluminum joints has been included in the introduction.** |

A Scientific explanation of the obtained results has been refined and ameliorated up to a fervent extent. Results are enumerated, test methods are utterly described, and interpretations have been correlated with results and previous literature findings. The overall summary should indicate the progress of the research and its limitations.

**Note:** All the necessary changes/added sentence has been shown in **BLUE COLOUR**.

Thank you very much in advance for taking your time in reviewing this manuscript.

Sincerely, we hope you will find our revision satisfactory.

Thanks, in anticipation.

Regards,

Dr. Shubham Sharma

(Corresponding author)
